# A Recent Lecture

**Published:** 1920-04-10

**Authors:** 


### DISEASES OF THE PANCREAS.

### A Recent Lecture.

An extremely valuable lecture upon the diagnosis of pancreatic disease was delivered by Sir Archibald Garrod at the London Hospital Medical College, and the full text published in a recent number of the *Lancet* (April 3). No more complete historical and medical survey of these conditions could have been made, and, coming from so great an authority, it provides a classic description of knowledge on the subject as it stands to-day. The lecturer, taking exhaustively each aspect of the problems involved, gave to both the research worker and the consulting physician of the future many invaluable leads, but it is possible that some of the simpler truths, which are of such paramount importance to the humbler practitioner, may tend to be lost in simply reading the lecture in its complete form, and we therefore venture to review some of those essential points which may be found useful in average medical practice.

### Diagnostic Indications.

The main diagnostic indications can be considered in three main groups. In the first come simple signs and symptoms such as tumour, pain, tenderness, vomiting, and cyanosis, or the result of pressure on neighbouring structures. Secondly, one may detect signs of failure in the external secretion, defective protein, fat, or carbohydrate digestion. And, thirdly, there may be found results of insufficient internal secretion, among which glycosuria is the most outstanding. The two syndromes associated with carcinoma of the head of the gland, and the condition known as bronzed diabetes are also to be remembered, but on this section of the lecture the most important advice is to be derived from the author's demonstration of the multiplicity of "pancreatic tests" and their inability to provide us with a "penny-in-the-slot" diagnosis.

The clinical picture must always be considered as a whole, and, with this fact driven home, the lecturer proceeds to devote his early attention to the finer points and pitfalls possibly associated with an attempted bedside diagnosis. The classical signs need not be detailed here, but several new appreciations are brought to light, and may advisedly be quoted.

### The Physical Signs.

If an enlarged pancreas be suspected it should be noted that the natural dulness on abdominal percussion may be obscured by overlapping stomach and intestine and the pancreatic tumour most difficult of detection with certainty, but it is maintained that percussion over the back allows the size of the gland to be mapped out with considerable accuracy. Pain also across the back is stated to be a much more constant and important symptom than is commonly appreciated—a point of value in distinguishing pancreatitis from intestinal obstruction, in both of which conditions vomiting may be severe and constipation obstinate. The degree of positive abdominal rigidity is also important in making this distinction; the pancreatic condition, although producing great distension, does not typically produce a marked rigidity.

Of the pressure results jaundice is by far the most conspicuous, and its mechanism is readily understandable, but, as Mayo Robson has shown, there exists a close connection between gall-stone troubles and interstitial pancreatitis, and from the anatomical relations of the biliary and pancreatic ducts an infection of one is likely to spread to the other, so that a catarrhal rather than pressure origin might be assigned to the sequence. Direct pressure may, however, with obvious results, affect neighbouring structures, such as the duodenum, portal vein, vena cava, or even the sympathetic nervous system at the great abdominal ganglia and plexuses.

The last of these leads us, in fact, to a new conception of certain so-called "thyroid" cases experienced during the war, and probably much commoner in civil practice than we are ordinarily disposed to remember. At least we have learned to look for signs of hyperthyroidism in conditions not formerly connected with the thyroid gland, and recently several observations have suggested that when thyroidal and pancreatic symptoms are seen in association it may be that the latter, and not the former organ is primarily at fault. As the lecturer suggests, "If in pancreatitis the eye symptoms referred to are due to excessive thyroid secretion, it is the sympathetic nervous system which is stimulating the gland to over-activity, and it is not wonderful that the sympathetic should be disturbed when the pancreas is the seat of disease, seeing how near a neighbour it is to the great abdominal ganglia and plexuses. Indeed, many of the symptoms of acute pancreatitis, the severe and often paroxysmal pains, the vomiting, and collapse are by many attributed to this proximity."

### Absence of External Secretion.

Among the "external secretion" tests come the most valuable aids to diagnosis that we possess, and they may therefore be considered in some detail. The pancreas is exceptional in dealing with all three classes of foodstuffs, but, in the lecturer's opinion, the signs of failure of digestion of fats are the most significant of all.

"If there be any single sign which, standing alone, may be regarded as pathognomonic of disease of the pancreas, it is true steatorrhœa. By this is meant the passage with the fæces of liquid fat which solidifies on cooling." More complicated estimates, such as the proportion of fat taken in the food which is lost in the fæces, or the degree to which the fat content of the stools is split into fatty acids and soaps, provide valuable means of gauging pancreatic efficiency, but in practice we must first be on the lookout for a conspicuous failure

**Diseases of the Pancreas**—(*continued*).

to split fats as giving excellent evidence of a pancreatic lesion. "In the stools of a healthy man some 75 per cent. of the fat is in the split form, whereas some patients with pancreatic disease pass as little as 20 per cent. in the forms of fatty acids and soaps. The great diagnostic significance of true steatorrhœa arises from the fact that it indicates not only an excess of fat in the stools, but also an undue proportion of *neutral* fat. In cases with no gross steatorrhœa the microscope may show abundant fatty globules and many acicular crystals of fatty acids. Moreover, the large bulk of the fatty stools may arrest attention, and in some cases they are justly described as elephantine."

### Tryptic Digestion.

Of the tests which aim at determining this portion of pancreatic power, two may be mentioned. It has been shown that a sample of the duodenal secretion can be obtained by a suitably constructed duodenal sound, or, better still, by Einhorn's ingenious device of a small metal capsule encased in gelatin and attached to a string or to an indiarubber tube. The capsule is swallowed, passes on through the stomach, and its progress may be checked by $x$-rays. As much as 5-10 c.c. of duodenal juice may be obtained, and its tryptic powers directly estimated on gelatin plates. The fallacy, of course, arises that whereas finding trypsin affords positive evidence of pancreatic activity, the failure to find it gives far less definite indication.

An excellent test by Oskar Gross is most often employed. An alkaline extract of fæcal material is made and its digestive action on casein estimated. Failure to obtain a positive action offers strong evidence of pancreatic inefficiency, although in this case a marked digestive effect may be produced by ferments other than trypsin, so that the converse does not hold. Actually, in review of these and sundry digestive messenger methods, the lecturer holds it to be "preferable to rely rather upon such gross phenomenon . . . than upon somewhat uncertain responses to these messenger tests."

### Carbohydrate Digestion.

Much the same applies to the real value of tests concerning carbohydrate digestion. Briefly put, the situation is that a diminished output of diastase in the stools and an increase of that enzyme in the blood and urine are common phenomena in cases of lesions of the pancreas; a sort of colourless jaundice, in which there is a direct escape of diastase into the blood. The results of diastase estimations are, however, not sufficiently reliable to be of great practical value, except under hospital and observational clinic conditions, and of considerably greater importance is the result of failure of the *internal* pancreatic secretion. Realising the direct connection between the glands efficiency and the phenomenon of glycosuria, let us briefly review the practical limitations in directly applying our knowledge to diagnosis. It is probable that in all instances of glycosuria the pancreas is purely to blame, although the main trouble may not be situated in the gland at all. The endocrine balance may be disturbed, as in the case of glycosuria, with excessive thyroid activity. In many ways may the pancreatic disturbance arise as a secondary effect, and, in contrast, we should remember that a small residue of pancreatic tissue may avert glycosuria, so that a patient in whom the gland is in reality grossly damaged may give no indication of excess sugar in the urine. Indeed, it may be said that, except in cases of chronic pancreatitis, glycosuria is an uncommon symptom in pancreatic disease. "Speaking generally, the absence of glycosuria affords no argument against the presence of disease of the pancreas, but in not a few cases its presence supplies the crowning evidence in support of a diagnosis based on other findings."

### The Nature of the Lesion.

The final discussion of exact pathology of the lesion in any particular case is one which, in a disease so difficult of elementary diagnosis, need not occupy us in this note. We have briefly abstracted certain points of the available methods which Sir Archibald Garrod described, and in leaving the subject at a point where a disappointing yet hopeful incompleteness appears to be the prevailing tone we cannot do better than quote his own words: "In this survey of the methods at our disposal for the diagnosis of lesions of the pancreas I have presented to you the separate pieces of a jig-saw puzzle. Only when the separate pieces have been fitted together can we form a distinct clinical picture of pancreatic disease. Nor must we forget that when we have determined the seat of a lesion we have attained to only half of a diagnosis. We need to diagnose the nature of the lesion also, and as it happens this is often, as in the conditions under discussion, the less difficult problem of the two."

